# Sex Differences in the Prevalence of Pain Trajectories Over a 14-Year Period in the Mexican Health and Aging Study

**DOI:** 10.1093/geroni/igaa057.1637

**Published:** 2020-12-16

**Authors:** Sadaf Milani, Martin Rodriguez, Rafael Samper Ternent, Rebeca Wong

**Affiliations:** The University of Texas Medical Branch at Galveston, Galveston, Texas, United States

## Abstract

Pain increases with age, disproportionately affects females, and is a major contributor to decreased quality of life. Because pain is dynamic, trajectories are important to consider. However, few studies have looked at longitudinal trajectories of pain, by sex, using population data. We used data from the 2001, 2003, 2012, and 2015 waves of the Mexican Health and Aging Study, a nationally representative sample of Mexicans aged 50 and older. Individuals who had direct interviews at each time point and complete information on analytical variables were included (n=5,135). Pain reported at these time points was used to define pain trajectories as persistent pain, development of pain, diminishing pain, fluctuating pain, and never pain, by sex. Multinomial logistic regression was used to assess the association of sex with pain trajectories, adjusting for baseline demographic, behavioral, and health factors. Females had a higher prevalence of persistent pain (25.1% vs. 11.5%) while males had a higher prevalence of never pain (35.8% vs. 22.2%). Development of pain, diminishing pain, and fluctuating pain was similar between males and females (14.4% and 14.6%; 14.8% and 15.8%; 23.9% and 22.4%, respectively). Females had higher odds of persistent pain (OR: 2.13; 95%CI 1.67-2.70) and development of pain (OR: 1.40; 95%CI 1.11-1.75) compared to males. Overall, females had a much higher burden of persistent pain compared to males. Understanding trajectories of pain in later life and life course mechanisms for this large sex difference observed is crucial to improve quality of life.

